# Prospects for dedicated energy crop production and attitudes towards agricultural straw use: The case of livestock farmers

**DOI:** 10.1016/j.enpol.2014.07.009

**Published:** 2014-11

**Authors:** P. Wilson, N.J. Glithero, S.J. Ramsden

**Affiliations:** Division of Agricultural and Environmental Sciences, School of Biosciences, University of Nottingham, Sutton Bonington Campus, Nottingham LE12 5RD, UK

**Keywords:** Bioenergy, Livestock farmers, Marginal land

## Abstract

Second generation biofuels utilising agricultural by-products (e.g. straw), or dedicated energy crops (DECs) produced on ‘marginal’ land, have been called for. A structured telephone survey of 263 livestock farmers, predominantly located in the west or ‘marginal’ upland areas of England captured data on attitudes towards straw use and DECs. Combined with farm physical and business data, the survey results show that 7.2% and 6.3% of farmers would respectively consider growing SRC and miscanthus, producing respective maximum potential English crop areas of 54,603 ha and 43,859 ha. If higher market prices for straw occurred, most livestock farmers would continue to buy straw. Reasons for not being willing to consider growing DECs include concerns over land quality, committing land for a long time period, lack of appropriate machinery, profitability, and time to financial return; a range of moral, land quality, production conflict and lack of crop knowledge factors were also cited. Results demonstrate limited potential for the production of DECs on livestock farms in England. Changes in policy support to address farmer concerns with respect to DECs will be required to incentivise farmers to increase energy crop production. Policy support for DEC production must be cognisant of farm-level economic, tenancy and personal objectives.

## Highlights

• Survey of English livestock farms determining attitudes to dedicated energy crops.
• 6.3% to 7.2% of surveyed farmers would consider growing energy crops.
• Limited potential for dedicated energy crops on livestock farms in England.
• Livestock farmers would continue to buy straw, even at higher market prices.
• Wide range of reasons given for farmers’ decisions related to energy crops.

## 1 Introduction

Renewable energy policies have become embodied legislation in a number of countries (e.g. the EU, Directive 2009/28/EU EU, 2009) as part of the drive to reduce reliance upon fossil fuels and mitigate greenhouse gas emissions (Goldemberg, 2007). While first generation biofuels (typically derived from crops which can be processed into food or energy [e.g. cereals, oilseed, sugar crops] Lovett et al., 2014) initially gained wide political support (Boucher, 2012), concerns over their legitimacy (Upham et al., 2011) and increasingly negative media coverage (Sengers et al., 2010) quickly surfaced. These concerns included food versus fuel land use change (LUC) (Boucher, 2012, Rathmann et al., 2010), indirect land use change (iLUC) (Kim et al., 2009) and the potential for biofuel induced land use change to lead to increased greenhouse gas emissions (Searchinger et al., 2007). Consequently, interest emerged in advanced, or second generation, biofuels that can make use of waste streams and co-products (e.g. corn stover, cereal [wheat, barley, rice] straw), or dedicated energy crops (DECs, e.g. miscanthus, short rotation coppice willow [SRC]). Hence second generation biofuels utilise biomass that is derived from non-food crops with greater energy generation efficiency (Lovett et al., 2014) or waste/co-product biomass. A commercial second generation processing plant now exists in the EU, in Italy (Anon, 2013), with development plans for other second generation plants, for example in the USA and Europe, already in place (Walker, 2013). However, in light of LUC and iLUC concerns, recent literature distinguishes between co-product (e.g. cereal straw) second generation biofuels (CPSGB) and dedicated energy crop second generation biofuels (DESGB; Glithero et al., 2012), providing clarity between feedstock sources used within different second generation biofuel supply chains. However, CPSGBs still have resource use implications that must be considered: straw is utilised in livestock bedding and feeding, soil conditioning, and nutrient provision for arable crops (Copeland and Turley, 2008, Glithero et al., 2013a, Glithero et al., 2013b, Powlson et al., 2011). Cereal straw is currently used within the UK in electricity power generation (e.g. the Ely Power Station) and recent research investment (e.g. BBSRC Sustainable Bioenergy Centre) has explored the potential to use cereal straw as a feedstock for lignocellulosic biofuel. With respect to DESGB, UK policies to encourage DEC production have until recently (August 2013) existed in the form of perennial bioenergy crop establishment grants (Natural England, 2013). However, despite the financial assistance that establishment grants provide, areas of these crops currently grown in the England are small and declining: SRC 2600 ha (declining from 6200 ha to 2600 ha over the 2008–2012 period), miscanthus 7000 ha (increasing from 7400 ha in 2008 to a peak of 9200 ha in 2009, followed by a decline to current levels) (Defra, 2013). It should be noted that these data are derived from non-National Statistics approved approaches and are additionally associated with large confidence intervals around the point estimates provided. However, these data do indicate that financial assistance alone, in the form of establishment grants, is insufficient to incentivise large scale production change. Moreover, the collapse of bioenergy companies that held contracts to purchase DECs has generated increased business uncertainty for those farmers willing to produce these crops (Sherrington et al., 2008) due to limited or non-existent alternative markets. This paper seeks to provide an understanding of English livestock farmer attitudes towards using their land for DEC production and their use of cereal straw when faced by an increased straw input price. This understanding will complement previous research for the arable sector in England (Glithero et al. 2013c) and be of direct relevance to policy makers seeking to achieve an increased supply of biomass production.

The rationale for examining attitudes of livestock farmers in part flows from calls to produce DECs on land not needed, or unsuitable, for food crops. Agricultural land use in the UK is dominated by both crop and livestock production. However, issues of land use appropriate for energy production represent global concerns, and are not restricted to a European or Western view alone (Fritsche et al., 2010, Zhuang et al., 2011, Tang et al., 2010). Previous studies have also considered the suitability of using ‘marginal’ land for energy crop production (McElroy and Dawson, 1986). However, defining ‘marginal’ land is potentially problematic; marginality can be defined in terms of economic output or reduced crop yield potential (e.g. Shortall, 2013, Gopalakrishnan et al., 2011), unsuitability for food crop production (e.g. Royal Society, 2008) or of low value for agricultural or biodiversity use (Royal Society, 2008). More structural definitions of land restrictions placed on energy crop production include excluding grade 1 and 2 land (the most productive for arable cropping) and land with slopes of >15% (Lovett et al., 2014, Wang et al., 2014). Swinton et al. (2011) question whether marginal land can be made available at sufficiently low cost, while Garnett (2009) argues that livestock farms on ‘marginal’ agricultural land may provide an important role in maintaining grasslands and the carbon sinks associated with these areas. Within the UK, grazing livestock production systems are predominantly located in western and upland areas (Fogerty et al., 2013, Harvey and Scott, 2013), where respectively higher annual rainfall and poorer quality agricultural land exists relative to the main arable cropping areas. Hence, while a standard definition of ‘marginal’ land does not exist, within the context of bioenergy production, the approach of considering agricultural land grades 3–5 as appropriate for bioenergy crops (Lovett et al., 2014) highlights the need to understand farm decision making within the livestock sector. Several authors have examined the environmental consequences of livestock production on marginal land (e.g. Acs et al., 2010, Oglethorpe, 2005) with respect to understanding livestock farmer behaviour and decision making in response to market and policy signals. To complement understanding of farmer behaviour in the arable sector (Glithero et al., 2013c), we therefore need a greater understanding of marginality with respect to livestock farmer decision-making and DEC production, particularly as livestock production is both an important component of the UK’s agricultural economy and its land use.

With respect to cereal straw, Glithero et al. (2013a) note the potential supply for larger scale use of straw in lignocellulosic processing facilities in England, estimating that 2.5 mt of cereal straw could be made available for bioenergy purposes. Such volumes of feedstock supply to biofuel uses will affect current straw markets (Glithero et al., 2013a), driving up product prices; the response of livestock farmers to this price increase is currently unknown but is of fundamental importance to any competing CPSGB industry, because the input feedstock cost is likely to form a substantial proportion of the overall costs of biofuel production.

Previous research examining farmer attitudes towards DEC production identified that availability of land (Adams et al., 2011), committing land to a single crop for a long time period (Glithero et al., 2013c), impact of DECs on land quality (Glithero et al., 2013c, Sherrington et al., 2008), relative financial return and cash-flow considerations (Adams et al., 2011, Glithero et al., 2013c), and knowledge of, or familiarity with, the crop (Glithero et al., 2013c) can have a direct impact upon farmer decisions about DEC production. Significant effects relating to managerial biographical factors (e.g. farmer age; Paulrud and Laitlia, 2010), managerial attitudes (e.g. objectives towards the environment; Augustenborg et al., 2012) and farm business physical factors (e.g. farm size and location; Paulrud and Laitlia, 2010) on attitudes towards DEC production have also been found. Conversely, other researchers have not identified significant relationships between farm and farmer characteristics and attitudes towards DEC production (Glithero et al., 2013c). Additional farmer attitude factors towards the production of DECs include the remoteness or location of their farm in relation to a bioenergy plant, the topography of their farm land, and prevailing climatic conditions that impact on soil moisture content (for both crop production and harvesting). The presence of farm advisors has been cited as a mechanism by which DEC production can be encouraged (Velandia et al., 2010, Glithero et al., 2013c, Alexander et al., 2014a) in an environment where lack of knowledge of the crops exist. With respect to quantifying the potential production of DEC within England, taking into account farmer willingness to consider growing SRC and miscanthus, either separately or jointly, Glithero et al. (2013c) estimate that arable farms in England could potentially supply 50,700 ha of SRC and 89,900 ha of miscanthus, assuming farmers would convert less than 10% of their land area. Other researchers have identified that farmers in the UK are only likely to convert a small proportion of the land area to DEC, and on their least productive land, even where interest in these crops exists (Sherrington et al., 2008).

Understanding both the factors that influence the supply of biomass feedstock and the competing demands for feedstock are therefore crucial to the development of a commercial bioenergy sector, in particular for second generation feedstock in its current embryonic commercial stage (Walker, 2013). By using survey techniques, this paper examines some of these potentially influential factors for livestock farmers in England. The specific objectives of this paper are to (a) describe the survey methodology adopted; (b) indicate the numbers of farmers willing to grow SRC and miscanthus and analyse the responses in relation to a number of farmer characteristics; (c) identify farmer attitudes and the main reasons given for growing and not growing these DECs; (d) estimate potential areas of these crops that could be grown on livestock farms in England based on the survey results and (e) draw national conclusions from these results in relation to the potential barriers/incentives identified to growing and not growing DECs and potential bioenergy supply. The survey methodology and design and results are outlined in 2, 3, respectively; this is put into the context of the UK bioenergy sector in Section 4. A summary, overall conclusions and policy recommendations are given in Section 5.

## 2 Methods

Building upon a body of attitudinal evidence that, for the arable sector, has captured attitudes towards straw production, straw incorporation/on-farm use or sale, and willingness to consider growing energy crops (Glithero et al., 2013a, Glithero et al., 2013b, Glithero et al., 2013c), a telephone survey was undertaken to capture data on livestock farmers’ attitudes towards DEC production and their responses to an increased price for cereal straw. The survey was conducted in England on three livestock farm types: dairy, less favoured area [LFA] grazing livestock and lowland grazing livestock.^1^ These farm types dominate land use in the upland and western regions of England and on grazing land (permanent pasture, temporary grassland and rough grazing) (Fogerty et al., 2013; Harvey and Scott, 2013). Taken together with evidence from previous research (Glithero et al., 2013a, Glithero et al., 2013b, Glithero et al., 2013c) conducting the survey on these three farms types will capture data for the majority of the agricultural land use area in England. In order to ensure direct comparability of data with previously published results for the arable sector in England, the questionnaire drew upon the design of previous research (Glithero et al., 2013a, Glithero et al., 2013b, Glithero et al., 2013c). Specifically, information was gathered on: (i) attitudes towards straw use given increased purchase price for the product (closed question responses to eight possible options); (ii) whether farmers would consider growing the DECs miscanthus and SRC at the present time (closed question: Yes, No, Already Growing; for each crop); (iii) factors that are important in their attitudes towards DEC production (closed question: [selecting all options that apply]); (iv) the percentage farm area on which they would be willing to grow DECs (closed question: percentage of farm area, for each crop); (v) importance rankings for key objectives relating to their farm and farm business (ranking questions: 5 point scale); and (vi) additional comments that the farmer wished to convey in relation to DECs or use of straw for bioenergy purposes (open question). Further details on the individual questions are detailed below in the description of methods of analysis. The survey was carried out in conjunction with the Farm Business Survey (FBS) by Rural Business Research ROs (Research Officers) between December 2012 and February 2013. The English FBS consists of a sample of approximately 3% of farm businesses across all regions of England, stratified to match the population of farm businesses, with a minimum standard output from their business activities of €25,000, across farm types defined by the cropping and livestock activity on the farm, and business size. Farm businesses are invited to take part in the FBS based upon randomly selected farm business address details supplied by Defra. The stratification for the FBS is based upon the annual June Survey returns (Department for the Environment, Food and Rural Affairs), and permits data aggregation, via population weighting, to estimate national production and financial returns for the sector. Within the FBS ROs visit the farm businesses that take part, on an annual basis, to collect financial and physical data relating to their farm and business activities. The surveyed farms for this study were a sub-sample of the full FBS sample (approximately 31% of the livestock farm types detailed above) across eight Government Office Regions (GORs) of England and two business size groups per farm type category, stratified by sample numbers within the FBS. Crucially, this sampling procedure ensured that the farms surveyed in this study were unbiased with respect to their opinions towards the production of energy crops, which overcomes any potential sampling bias when undertaken more general random sampling approaches, for example, via postal surveys. The sub-set of FBS farm businesses which took part in this telephone survey were randomly selected from the FBS sample for the farm types of interest. Each respondent invited to take part in the telephone survey was previously known to the RO who conducted the survey. This respondent-researcher relationship was crucial to ensuring that all of the 263 respondents who were invited to take part completed the telephone survey; hence respondents neither agreed nor declined to take part because they had either a particular interest, or no interest, in the survey topic (e.g. DECs). The number of farms surveyed in each farm type and GOR is given in Table 1. Reflecting the geographical nature of livestock production in England, the South West region of England represents the largest number of sample returns (31%), followed by the North West (19%); note that less than 4% of the sample were from the East of England.

**Table 1:** Number of survey respondents by farm type and government office region.

GOR	Dairy	LFA grazing livestock	Lowland grazing livestock	*GOR total*
North East	3	19	5	*27*
North West	16	21	12	*49*
Yorkshire and the Humber	9	10	4	*23*
East Midlands	14	8	8	*30*
West Midlands	9	4	8	*21*
East of England	5	0	5	*10*
South East	6	0	15	*21*
South West	36	17	29	*82*

*England total*	*98*	*76*	*86*	*263*

Following the approach of Glithero et al. (2013c) and drawing upon data held about the farm in the FBS, responses relating to willingness to grow SRC and miscanthus (Yes, No; per crop) have been analysed with respect to categories for: farmer age (years: under 50; 50–64; 65 and over); EU England region location (North, East or West); land ownership (greater than 50% area owned; less than 50% owned); farm type (dairy, LFA grazing livestock, lowland grazing livestock); farm size (large, medium, small; as defined by FBS); and farmer educational attainment (school level only [GCSE’s, A-levels, Apprenticeships and other]; college level; university level [degree or postgraduate]). Chi-squared tests were undertaken to test the hypothesis that there is no significant relationship between each of the above factors and farmer attitudes towards growing SRC and miscanthus. Where farmers noted a willingness to grow SRC or miscanthus (or both), data were obtained on the percentage land area (per crop) that they would consider growing across the following categories (up to 10%; >10–25%; >25–50%; >50–75%; >75–99%; 100%).^2^ Combining data held about the farm’s utilised agricultural area (UAA) from the FBS, with the responses to percentage farm area that the farmer would be willing to grow, minimum and maximum crops area per farm were calculated by taking the lower and upper bounds of the above percentage categories (lower bound of up to 10% was assumed as 0%). These data were then aggregated to national (England) levels following a standard aggregation weighting procedure (see Glithero et al., 2013b) which calculates aggregation weights as the Defra June survey total farmed area per farm type, per GOR divided by the survey farm area per farm type, per GOR; the individual farm data are then weighted by this aggregation factor and estimates of potential crop production across the eight GORs of England estimated. Minimum and maximum potential regional and national supply estimates of DECs were then calculated.

Once farmers had indicated their willingness, or otherwise, towards growing SRC or miscanthus, they were asked to select which factors were important in their decision making across a range of practical, environmental, financial and knowledge factors. Practical factors consisted of: lack of appropriate machinery; use of known machinery; ease of crop management; committing land for a long period of time; ‘need planning permission from landlord’. Environmental factors examined were: positive environmental impact (of crop); negative environmental impact; Nitrate Vulnerable Zone (NVZ) restrictions; ‘land quality aspects’. Financial and knowledge factors examined were: time to financial return for crop; market for crop; no market for crop; profitability; local working example; no local working example. These responses were then analysed with respect to willingness to grow the energy crop or not, for each crop of SRC and miscanthus, to indicate the percentage of responses per importance factor. In addition to the pre-determined factors within the questionnaire, farmers could also add additional factors that were important in their decision making; these factors were grouped together in broad categories with specific comments detailed as issues raised by farmers in the survey.

In order to capture data on potential responses to an increased purchase price of straw, farmers were asked to indicate if, and how, they would change their practices towards straw purchase and use in light of a change in market conditions for straw. Farmers were provided with a realistic practical business scenario of an increase straw price of £100/t for ‘big square bales’ (prevailing market price at the time of the survey was approximately £40/t). Farmers were asked to select from a range of responses encompassing increasing own cereal crop production, start own cereal crop production, change livestock bedding material from straw to another product, change from loose housing to cubicles to reduce or remove the need for straw, continue to buy in straw, reduce livestock production, stop livestock production, and changing to grow taller cereal varieties (for additional straw). In addition, farmers could indicate other options as responses to this scenario. The percentage responses to these factors were calculated by farm type groups.

## 3 Results

The livestock farmers surveyed were asked if they would be willing to consider growing SRC willow and miscanthus, requiring separate responses for each crop; farmers could respond with ‘yes’, ‘no’ or ‘already growing the crop’. In total from the 263 responses received, 19 farmers (7.2%) indicated they would consider growing SRC willow, while 17 (6.3%) noted a willingness to consider growing miscanthus; 12 farmers (4.6%) indicated a willingness to grow both crops. None of the farmers surveyed were already growing either of these two crops. Using Chi-squared tests, responses were analysed to test the hypotheses that there was no significant relationship between farmers’ age, farmers’ education, farm ownership, farm type, farm size and farm EU region location to the farmer response to attitudes towards growing SRC or miscanthus. These hypotheses were accepted for all factors at the 95% level of significance. However, it is informative to note that at the 90% level of significance land ownership (greater or less than 50% of farm area owned) has a significant impact on response; farmers with greater than 50% farm area owned are more willing to consider growing SRC (9.6% compared with 4.3% for farmers with less than 50% owned area) and miscanthus (8.9% compared with 3.4% for farmers with less than 50% owned area). Additionally, at the 90% level of significance, there is a significant relationship between EU region and willingness to consider growing miscanthus (EU East region more willing to consider growing miscanthus 13.1% compared with 4% EU North, 4.8% EU West): *p*-values for these results are given in Table 2.

**Table 2:** *p*-Values from the Chi-squared tests.

		SRC	Miscanthus
Farmer age		0.167	0.163
Location	EU region	0.342	0.053
Land ownership		0.097	0.072
Farm	Type	0.541	0.427
	Size	0.722	0.491
Education level		0.730	0.422

**Fig. 1:**
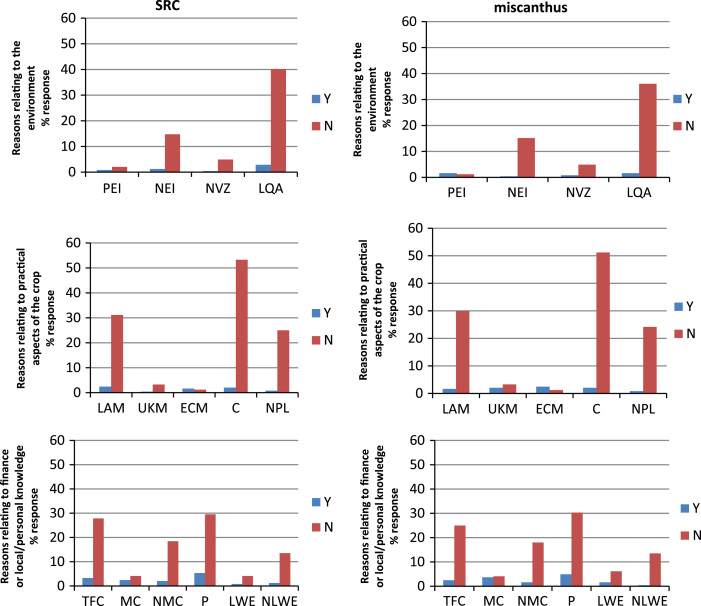
Percentage responses from those that would and would not be willing to grow short rotation coppice (SRC) and miscanthus. PEI positive environmental impact, NEI negative environmental impact, NVZ nitrate vulnerable zone restrictions, LQA land quality aspects, LAM lack of appropriate machinery, UKM use of known machinery, ECM ease of crop management, C committing the land for a long time period, NPL needing permission from landlord, TFC time to financial return on crop, MC market for crop, NMC no market for the crop, P profitability, LWE local working example and NLWE no local working example.

Fig. 1 presents reasons given for being willing or not willing to consider growing SRC or miscanthus following the approach of Glithero et al. (2013c). For both SRC and miscanthus common issues emerge as reasons cited for deciding to not consider growing these crops. Within the practical reasons, ‘committing land for a long period of time’, followed by ‘lack of appropriate machinery’, are consistently cited. In addition, for approximately 25% of respondents, ‘permission from the landlord’ would be required. Within the practical reasons grouping, the ‘use of known machinery’ and ‘ease of crop management’ were cited by very small numbers of respondents with respect to either being willing or not willing to consider growing these crops. With respect to environmental reasons the major issue cited relates to ‘land quality aspects’, with damage to drains and cost of changing land back to agricultural use identified to respondents as the definition for this category. Approximately 15% of respondents cited ‘negative environmental impacts’ as a reason for choosing not to consider growing either crop. ‘Nitrate Vulnerable Zone restrictions’ were not a key determining factor for farmers, and very limited numbers of respondents cited ‘positive environmental impact’ of miscanthus or SRC as an important aspect in their decision making. Profitability was cited as a key financial driver against considering growing these crops, followed by ‘time to financial return’ and ‘no market for the crop’. It is informative to note that while ‘no local working example’ was cited by over 10% of farmers as a reason for not being willing to consider growing either of these crops, the presence of a ‘local working example’ has also been cited by a small number of respondents as a reason for *not* being willing to consider growing either crop. From the responses citing willingness to consider SRC or miscanthus, ‘profitability of the crop’ is highlighted as a key influence, followed by ‘market for the crop’ and ‘time to financial return’. Note however, that the modest number of positive responses towards growing the crops leads to single responses having a relatively large influence on the overall percentage influence recorded. Respondents also provided additional comments to the pre-set potential responses. One hundred and thirty one (49.8%) additional comments were recorded. These have been categorised in Table 3 as ‘interest and morality’ (11 responses), ‘current and future farming/business activities’ (19), ‘land resource availability’ (32), ‘land quality/topography’ (41), ‘knowledge’ (14), ‘other’ (14). It is informative to note the large number of comments relating to land quality/topography which frequently highlighted aspects of unsuitability of land for crop production, steepness of slopes or wet land and weather conditions.

**Table 3:** Additional comments for not growing short rotation coppice and miscanthus (131 comments)

Segment		Typical comments—summarised	Selection of quotes
Interest and morality	11	• Not interested • Moral point towards using land for food production	“Simply not interested in growing either crop”
			“Not interested in diversifying to bioenergy crops, would rather focus on business as it currently runs”
			“Should be producing agricultural produce as land best suited for that”
			“Not sustainable, think it’s a fad”
Current and future farming/business activities	19	• Doesn’t fit with current activities • Desire to continue/expand current agricultural production • Planning towards retirement	“Farm focusses on livestock production rather than arable, no interest in bioenergy crops”
			“Son joined partnership and aim to increase production with large investment in place so no thought of reducing agricultural output”
			“Too old to consider long term commitment”
			“Currently running very profitable low labour simple grass based system. Don’t need any complications”
Land resource availability	32	• Not enough land for bioenergy crops • All land needed for current activities • Would have to reduce other production due to land constraint	“Farm needs all land to support dairy herd so no area to grow energy crops”
			“Need grass”
			“Not prepared to give up valuable forage land”
			“Not enough land to spare. Currently rent in a lot for own livestock needs”
Land quality/topography	41	• Land too productive for energy crops • Poor quality land only suitable for grazing • Hill land or steeply sloping • Wet land and wet weather conditions	“This is a hill farm, no land to grow this type of crop”
			“Farm all permanent pasture with some SDA [Severely Disadvantaged Area] land”
			“Land not suited for bioenergy crop production - hard enough getting quality forage to grow”
			“Land is largely inaccessible, better suited to grazing and a bit of hay making.”
			“Topography of farm [steep slopes] eliminates all possibilities of growing these crops”
Knowledge	14	• Distance to energy processing plant • Lack of knowledge of this crop • Knowledge of companies not fulfilling contracts to buy crops	“Don’t know anything about husbandry for this”
			“Too far away from power station”
			“Used to grow Miscanthus but found it unprofitable and little local market”
			“There is a local market for the crop but local experience has shown that they do not always buy available crops”
			“Neighbouring farms have SRC and have heard bad reports about lack of profitability and management”
Other	14	• Tenancy/landlord constraints • Conflict with current environmental schemes	“Cannot plough—in ESA [Environmentally Sensitive Area] scheme”
			“All land is situated within a National Park”
			“Land committed to HLS [Higher Level {environmental} Scheme] and Organic schemes”
			“Duration of tenancy”

**Fig. 2:**
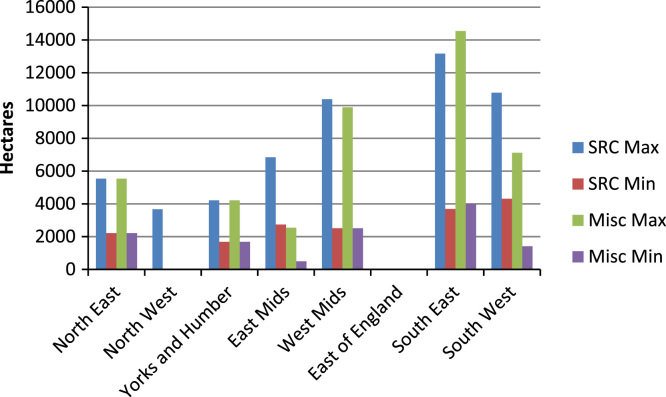
Potential maximum and minimum production of short rotation coppice (SRC) and miscanthus (Misc) from dairy, LFA grazing and lowland grazing farms by Government Office Region of England based upon respondents who would consider growing each crop.

Those livestock farmers willing to consider growing SRC or miscanthus were asked to indicate the percentage of their farm area that they would be willing to commit to these crops. These responses were then combined with data from the FBS on farm area, and aggregated to provide GOR and hence national (England) estimates of the minimum and maximum crop areas that would potentially be grown on livestock farms. Fig. 2 presents the results of this analysis; the largest potential maximum crop growth area for both SRC and miscanthus is the South East of England. More modest areas of crop production possibilities were identified in the North East, North West, and Yorkshire and the Humber. For England, the maximum (minimum) potential area of SRC that would be grown on dairy, LFA grazing livestock and lowland grazing livestock farms is 54,603 ha (17,156 ha); the respective results for miscanthus are 43,859 ha (12,321 ha).

**Fig. 3:**
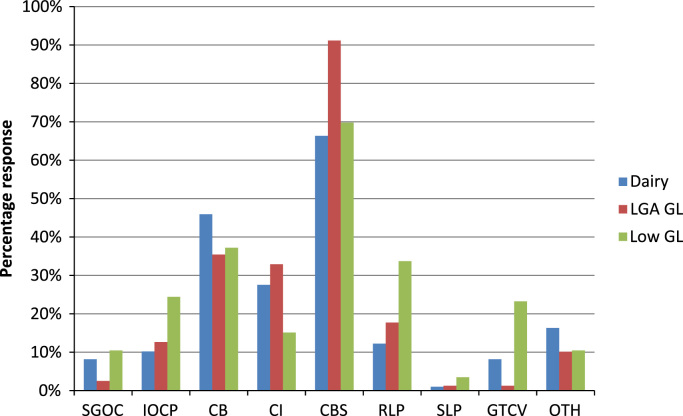
Livestock farmer responses to possible strategies for responding to an increased straw price (to £100/t) by farm type. Key: SGOC=start to grow own cereal crops for straw; IOCP=increase own cereal crop production; CB=change bedding from straw to another product; CI=change infrastructure from loose housing to cubicles; CBS=continue to buy in straw; RLP=reduce livestock production; SLP=stop livestock production; GTCV=grow taller cereal varieties; OTH=other. LGA GL=LFA grazing livestock; Low GL=lowland grazing livestock.

Livestock farmers were asked to consider how they would respond to an increased price of straw as detailed in the methodology section; on a number of farms a negative response was recorded as “Not Applicable” for specific possible responses, for example where farmers viewed starting own crop production, increasing crop production or growing taller varieties, as not applicable to their farm or business situation. Fig. 3 provides the percentage of “yes” responses to strategies for responding to the increased straw price question, across the three farm types examined; significant differences in the number of positive responses to the strategies by farm type groups were found (Chi-squared test, *p*-value <0.001). Key results demonstrate that across all farm type groups the majority of farmers would not stop keeping livestock in response to this increased price of straw, as noted by less than 5% in each farm type group choosing this option. In response to increased straw prices, most livestock farmers indicated that they will continue to buy straw. However, lowland grazing livestock farmers are more likely to reduce livestock production given increased straw prices; this response may in part be driven by the greater flexibility in agricultural and land-use production possibilities that lowland grazing livestock producers have in comparison to dairy or LFA grazing livestock farms. With respect to increasing the production of ‘own’ cereal crops, lowland grazing livestock farmers are the group most likely to undertake this option. Changing the bedding from straw to another product was a more popular response than changing infrastructure (e.g. from loose housing to cubicles).

## 4 Discussion

Our findings demonstrate that only modest numbers of livestock farmers are willing to consider growing DECs (7.2% and 6.3% for SRC and miscanthus, respectively); this is lower than observed for arable farmers in England (11.9% and 17.2% for SRC and miscanthus, respectively; Glithero et al., 2013c) and is argued to flow from a range of geographic, land tenure, land quality and financial considerations as explored further below. However, a central outcome from the results presented is that the lower level of interest shown from livestock farmers with respect to growing DECs represents a substantial challenge to policy makers seeking to encourage DEC production, in particular from geographic areas that are characterised as being more agriculturally marginal. In part the difference between livestock and arable farmer willingness to grow DECs may be explained by the proximity of current concentrations of markets for DECs, where greater concentrations of land use for DEC production are found within the East Midlands, Yorkshire and Humberside and the South West (Lovett et al., 2014); while the latter region is predominantly livestock based, the former regions are predominantly arable and represent areas where farmers may have been more exposed to the possibility of growing these crops via information from their farmer neighbours, or from companies seeking to secure crop supplies. It is informative to consider the implications of these broader geographic regional findings. The combination of greater interest in growing DECs from arable farmers, and the greater interest from livestock farmers in the EU East region of England, together indicate that developments in the bioenergy sector that rely upon DECs or cereal straw should be located in the East of England (Glithero et al., 2013a). While this may contrast with calls for DEC production on ‘marginal’ land, it will result in bioenergy facilities being located in areas where supply of both cereal straw and DECs have greater potential. For livestock farmers, land ownership influences decisions about DEC production, with those farmers owning more than 50% of their land area being more willing to consider growing DECs; in part these land ownership influences reflect restrictions placed upon tenants by landlords who wish to maintain particular types of livestock based land use activities on their land. Livestock farmers in the EU East region of England are more likely to consider growing miscanthus than livestock farmers in the other EU regions of England; this is possibly a reflection of the more marginal nature of livestock production in the east, where lower precipitation levels limit grass forage production and hence increase the profitability of crop production relative to grazing livestock enterprises. The land ownership and geographic influences observed for livestock farmers contrast with those for arable farmers in England, where land ownership and regional influences were not found to have a significant influence on attitudes towards growing DECs (Glithero et al., 2013c); however, the results for livestock farmers presented here do reinforce findings for attitudes towards production of bioenergy feedstocks in other countries (e.g. switchgrass in Tennessee, Jensen et al., 2007; SRC on marginal land in Germany, Schweier and Becker, 2013). From the results presented above, issues relating to the impact of DECs on land quality, committing land for a long period of time, lack of appropriate machinery, relative profitability of the crop and time to financial return were cited as common reasons for not considering growing these crops, confirming attitudes reported in previous studies (e.g. Glithero et al., 2013c, Schweier and Becker, 2013, Sherrington et al., 2008, Swinton et al., 2011, Tate et al., 2012), and providing clear signals to policy makers that there are constraints and barriers to the take up of DEC crops by farmers. Some of these are actual: relative profitability of existing farm enterprises will vary from farm to farm and those farmers with healthy ‘enterprise gross margins’ (financial value of output less direct, variable costs of production) will be less willing to switch to DECs, other things being equal. However, some barriers to take-up may be more perceived than real - for example, the effect of DECs on land quality over time once established. For livestock farmers, land quality was also given as a reason for not starting production: topography, poor cropping ability of hill land, and ‘wet land’ were all cited as reasons for not considering growing DECs. Within the context of DEC production, these findings contrast directly with calls for DEC production on marginal land. Specifically, livestock farmers citing these concerns demonstrate that the ‘marginality’ of the land, with respect to use for mechanised crop production, makes the prospect of DEC production in these areas less viable, and hence less likely, than in ‘non-marginal land’ areas. The dichotomy between policy objectives that aim to minimise food versus food conflicts and the potential uptake of DEC production on English livestock farms, some of whom operate in challenging geographical and climatic conditions, is clearly evident here. Where livestock farmers were willing to consider growing SRC and miscanthus, ‘profitability’, having a ‘market for the crop’, ‘time to financial return’ and ‘ease of crop management’ were cited as important factors influencing their decision making. While representing small numbers of respondents in this study, these factors are of direct interest to policy makers seeking to incentivise DEC production. In particular, these farmers implicitly consider their land to be marginal with respect to the alternatives that they face within their own farm businesses. In some respects it would, from a policy perspective, be more productive to target these marginal farmers, rather than to attempt to identify, from a perspective external to the farm business, marginal land using a pre-conceived measure, such as soil quality or yield potential.

Placing the above points in wider context, it is informative to recognise that the results presented represent findings from a survey of stated attitudes at a single point in time towards a series of pre-determined questions. Moreover, the use of a telephone survey during the winter months may have influenced farmer responses. Previous authors have criticised such linear decision making models as not seeking to understand underlying motivations. Within economic geography approaches, Radwan and Kinder (2013) and Jones and Murphy (2011) argue that innovation or adoption of different practices are more complex than can be elicited from the approaches adopted within this study, and that adoption involves aspects such as learning, social norms and networks, all of which are dynamic. Within the specific case of energy crops, Alexander et al. (2014a) note that adoption is likely to follow a typical S-shaped curve over time, hence it is not directly possible to capture the dynamics of DEC supply in the future from the approach adopted here. However, survey approaches provide a succinct method of data capture across a large number of observations and have been used in previous research that has direct relevance to this current study (Glithero et al., 2013a, Glithero et al., 2013b, Glithero et al., 2013c). Moreover, Alexander et al. (2014b) note that within an agent-based modelling framework the first stage in the decision making process for farmers is appropriately captured by ‘willingness to consider growing the crops’.

Accepting the restrictions outlined above as caveat to the survey findings, we provide estimates of the national potential for DEC supply from these farm types which are argued to be of policy and industry relevance. Specifically, on the assumption that livestock farmers in England grew DECs in line with their stated willingness to consider growing these crops identified by the representative livestock farmers in this survey, the maximum (minimum) area of SRC that would be produced on livestock farms in England is 54,603 ha (17,156 ha); the respective results for miscanthus are 43,859 ha (12,321 ha). Glithero et al. (2013c) estimated a potential supply 50,700 ha of SRC and 89,900 ha of miscanthus from arable farmers in England. Taken together these data indicate potential for approximately 100,000 ha of SRC and 130,000 ha of miscanthus production in England. These estimates represent a small proportion of the estimated areas available for DEC production cited in previous studies (3.1 Mha for DECs, Haughton et al. (2009), 362,859 ha of miscanthus, Lovett et al. (2009), 7.3 Mha, Lovett et al. (2014)) reinforcing the finding that the constraint to DEC supply will be economic considerations rather than planning or structural constraints. With respect to encompassing farm-level economic considerations, Alexander et al. (2014a) estimate ‘maximum potential’ supply in England of 81,000 ha of SRC and 141,000 ha of miscanthus, with supply levels being output price sensitive. While Alexander et al. (2014a)’s area estimates are more directly in line with the results presented above, Alexander et al. (*op cit*) estimate DEC supply to largely occur in livestock dominated areas of England, with small crop areas likely to be found in the arable dominated regions. Moreover, Alexander et al. (2014a)’s estimate is based upon a comparison of conventional arable cropping with DEC production in all regions; in reality the agricultural activities in the main areas they identified for DEC production are predominantly livestock based, and farmer decision making will therefore be based upon a comparison of livestock production against DECs. However, it is informative to note that both the estimates from this current study, and from Alexander et al. (2014a) represent considerable increases from current DEC areas and would require changes in policy support (Glithero et al., 2013c) in order to achieve these levels of production. Such policy support will need to be cognisant of the range of issues identified by farmers as highlighted above. Previous studies (e.g. Alexander et al., 2014a, Glithero et al., 2013c) have noted the need for enhanced extension services to farmers in order to encourage DEC production. With respect to policy implications, Alexander et al. (2014b) note that the provision of establishment grants for DECs provides a cost effective GHG emissions abatement policy, and one which would be more effective in encouraging DEC uptake than providing higher subsidies to power generation companies. The 50% establishment grant for DEC in England closed in August 2013, without a clear indication of what may replace this (Alexander et al., 2014a); White et al. (2013) argue that consistency of policy rules are critical to development of renewable energy. Others have argued for area-based payments for the initial years of on-going energy crop production (Lindergaard, 2013).

With respect to tenant livestock farmers, policy incentives may need to be targeted towards landlords in order to allow tenants’ greater freedom in their land use practices and production possibilities. Policies developed in the absence of understanding these key issues are argued to be inherently less likely to achieve their desired aims. Noting the ‘marginal farmer’ concept outlined earlier; a potentially cost effective way to target these farmers would be some form of auction of contracts for the right to receive payments to grow DECs. In the context of contracts for agri-environmental policy, Latacz-Lohman and Schillizi (2005) suggest that auctions work best “when the number of bidders is high, contracts are homogeneous and landholders are heterogeneous in their compliance costs”. Useful experimental research could be conducted to test whether DEC contracts, and potential markets in these contracts, meet these criteria.

In response to a substantial increase in straw prices, the majority of livestock farmers indicated that they would continue to produce livestock, with lowland grazing livestock farmers being more likely than dairy or LFA grazing livestock farmers to consider reducing livestock production; the lowland grazing livestock group were also more likely to increase production of their own cereal crops and to consider growing taller varieties. These responses, in part, demonstrate the specialised nature of dairy production in comparison to lowland grazing production, and the more restricted range of production possibilities found in the upland and hill areas of England (Harvey and Scott, 2013). LFA grazing livestock farmers would overwhelmingly continue to buy in straw at high prices, while the majority of dairy and lowland grazing livestock farmers would also continue to buy in ‘high price’ straw. Changing bedding or livestock building infrastructure would also occur, but on a smaller proportion of livestock farms.

Livestock farmers may be more generally reluctant to consider alternatives to straw use or to substitute existing land uses for bioenergy feedstocks. It has been argued that this is because agricultural assets have low values outside of the industry (the phenomenon of ‘asset fixity’, see e.g. Boetel et al., 2007); existing patterns of production are likely to persist due to low asset resale values. This will particularly be the case where assets have very specific, non-transferable uses, as is often the case in dairy production. Disinvestment also reduces flexibility: in a survey in Germany, Musshoff et al. (2013) present evidence that farmers value the option to gain information on future cash flows by deferring disinvestment, even where it would be financially optimal to realise the (relatively low) value of the agricultural assets tied up in a particular aspect of the business, or the business as a whole. Capital asset realisation removes this option. Richards and Green (2003), in the context of perennial crops, refer to ‘hysteresis’, the continuation of a particular economic activity in agriculture in order to gain the small chance of worthwhile returns sometime in the future—essentially an attitude of ‘not wanting to miss out’. The corollary is that, once established, there would be an option value for continuing in crops such as miscanthus or SRC production.

A straw-based second generation bioenergy plant may lead to an increased amount of straw being made available to the market, as arable farmers increase the volumes of straw baled (Glithero et al., 2013a). However, our findings indicate that bioenergy processors will have to compete, in the market for cereal straw, with livestock farmers. Evidence from Germany, where large areas of oilseed and maize crops are grown for energy demonstrates that as land available for primary food production decreases, land prices and rents increase, in turn increasing the cost of biomass production (Lupp et al., 2014). Larsen et al. (2013) note that even with increased production efficiencies in Danish agriculture, demand for wheat straw for CPSGB production, would fully utilise wheat straw residues by 2030, in addition to limiting livestock production expansion. From the evidence presented here, English livestock farmers’ response to straw price increases would appear to be price inelastic; while some product substitution may occur, the importance of straw within livestock production would conceivably lead to these livestock farmers outcompeting bioenergy purchasers. The bioenergy sector will need to secure feedstock at a sufficiently competitive price to ensure commercial success; for livestock farmers, while the cost of straw represents an important production cost, other costs typically outweigh those incurred from purchasing straw. Moreover, in order for livestock farmers to achieve large scale reductions in straw use, investment in infrastructure (e.g. cubicle housing for cattle) will be required. Arguably only when relatively high straw price thresholds are breached, and are forecast to remain high, will farmers seek to make such investments.

Evidence from the results presented above, combined with previous evidence on straw availability (Copeland and Turley, 2008, Glithero et al., 2013a, Glithero et al., 2013b) indicates that under contemporary market, policy and regulatory conditions, wide scale UK straw-based bioenergy production will be constrained by biomass availability and feedstock price pressures. Moreover, attitudes towards DEC production (Glithero et al., 2013c, Sherrington et al., 2008) demonstrate a range of farm-level, tenancy and market condition factors that will additionally constrain large scale UK DEC biomass production. In addition, ‘marginal land’, around which some livestock production systems are based, has been demonstrated to be a constraint to the production of DEC, rather than a perceived opportunity which has been frequently assumed (Ajanovic, 2011, Fischer et al., 2010). Others have also recognised the constraints these geographical areas place on DEC production, hence limiting the sustainable supply of biomass (Bindraban et al., (2009)). However, both straw-based and DEC-based bioenergy do offer entrepreneurial opportunities to farmers. Recent UK Government incentives for renewable energy have led to increased farmer uptake of solar and wind power generation, and use of biomass boilers, in the UK; farmers have been incentivised by Government-guaranteed financial return, yet farmer attitudes play a central role to the uptake of renewable energy opportunities (Tate et al., 2012). Currently, DESGB production incentives are limited to financial assistance for crop establishment, while no Government incentives exist for CPSGB. As argued by Glithero et al. (2013c), further incentives will need to be provided to farmers to engage in supplying biomass feedstock on a larger scale. European farmers also face demands to increase the supply of ‘ecosystem services’, or ‘greening’ activities, within the requirements of the revised Common Agricultural Policy (CAP) (EU, 2011). These are required on predominantly arable farms, however, Defra’s (2014) implementation of the revised CAP means that miscanthus and SRC will not count as crops that provide environmental benefits under the ‘greening’ rules. Contrasting this outcome, Rowe et al. (2009) find that DECs of miscanthus and SRC can provide positive outcomes to biodiversity, soil properties and GHG mitigation when compared to arable crops, and these outcomes are also noted for miscanthus by Wang et al. (2014). The financial returns of primary food production have also increased in recent years in part because of the success of biofuel-related policy mechanisms (Wright and Cafiero, 2011). These improved food prices reduce the incentive for farmers to adopt new or alternative cropping strategies.

## 5 Conclusions and policy recommendations

While calls for the production of DECs on ‘marginal’ land have been made, much previous research has examined the suitability of land for DECs without examining issues related to the economics of agricultural production or farmer decision making on farms. Our findings demonstrate limited potential for DEC production on farm land in England and reinforce previous estimates of aggregate DEC supply potential (Alexander et al., 2014a). Moreover, we have identified that livestock farms will typically continue to buy in cereal straw at higher prices, with consequential impacts on the economic viability of any second generation bioenergy sector. This research highlights some key policy messages for those seeking to achieve a positive transition pathway towards the increased use of biomass for bioenergy purposes. Moreover, with respect to targeting biomass production policies at particular farm types, our results indicate that livestock farmers are marginally less interested than arable farmers (Glithero et al., 2013c) in growing DECs. With respect to potential policy implications for increasing biomass supply from farm land in England, our results lead to the following policy suggestions. Maintenance grants and on-going area-based payments for DECs would, in-part, address issues of the time taken to gain a financial return from these crops. Supporting DEC-specific extension services would increase farmer awareness of the ease of crop management for DECs. De-risking the output market for DECs through government-backed output contracts would provide a guaranteed market for the crop addressing a key issue raised. Incentivising DEC production via eligibility for energy subsidy payments (e.g. Renewable Obligations Certificates [ROC]) would improve the relative profitability of DEC in comparison to the current financial returns. Targeting both farmers and landlords with respect to policy messages may be required to overcome tenancy restriction faced by some farmers. Geographic targeting of support within a specific radius of a biomass plant location, or co-supporting biomass plants alongside farm-level biomass production may also be required. In the absence of such policy developments the combination of increased market prices for primary food products, and the policy environment in which farmers currently operate in the UK, will arguably further restrict biomass production from farm land. In addition, policy makers and the biomass industry must recognise that, in regions within, or in close proximity to, livestock production, farmers will potentially out-compete a biomass plant in the market for cereal straw. Hence, policy makers seeking to incentivise DEC production arguably need to re-think the current DEC support mechanisms to address profitability, market risk, land suitability and land quality concerns, combined with issues of lack of knowledge of DEC production amongst farmers in general, and the need for landlord permission on some tenanted farms. Policies supporting the production of DEC on lower grade agricultural land, or land with lower agricultural or biodiversity potential must acknowledge issues of economic, tenancy and personal objectives if they are to succeed. Moreover, if cereal straw is to play a central role in second generation biofuel production, policy makers may need to incentivise changes in livestock building infrastructure, to reduce livestock farmers’ demand for cereal straw. It is also essential that policy makers ensure that food, fuel and ecosystem services policies are integrated, rather than conflicting, in order to provide coherent and consistent policy signals to farmers.

## Footnotes

1 Farm types as classified in the English Farm Business Survey (Defra).

2 There were no responses that indicated a willingness to grow >50% of farm area in these crops.
